# Modification of Carbon Nanomaterials by Association with Poly(3-octylthiophene-2,5-diyl) as a Method of Improving the Solid-Contact Layer in Ion-Selective Electrodes

**DOI:** 10.3390/membranes12121275

**Published:** 2022-12-16

**Authors:** Barbara Niemiec, Robert Piech, Beata Paczosa-Bator

**Affiliations:** Faculty of Materials Science and Ceramics, AGH University of Science and Technology, Mickiewicza 30, PL-30059 Krakow, Poland

**Keywords:** ion-selective electrodes, carbon nanofibers, hierarchical nanocomposites, potentiometric sensor, hydrophobic material, high electrical capacity

## Abstract

A new group of carbon nanomaterials modified with poly(3-octylthiophene-2,5-diyl) for solid-contact layers in ion-selective electrodes was obtained. The materials were characterized by scanning electron microscopy and measurement of the contact angle. The modification greatly improved the hydrophobicity of the materials, and the highest contact angle (175°) was obtained for a polymer-modified carbon nanofibers/nanotube nanocomposite. The electrical parameters of the electrodes were determined using the methods of chronopotentiometry and electrochemical impedance spectroscopy. The highest electrical charge capacity was obtained for polymer-modified carbon nanofibers (7.87 mF/cm^2^). For this material, the lowest detection limit (10^−6.2^ M) and the best potential reversibility (SD = 0.2 mV) were also obtained in potentiometric measurements.

## 1. Introduction

The development of all-solid-state electrodes started when Cattrall and Freiser [1] presented a coated-wire electrode in 1971. This electrode was made of platinum wire, which was directly covered with an ion-selective membrane. This was the first electrode design to eliminate the internal solution that was an integral part of the earlier known classical ion-selective electrodes. The lack of internal solution allows the electrodes to be miniaturized and eliminates the problem of leakage of internal solution into the sample [2]. However, this solution also had some disadvantages. The greatest of these was the lack of potential stability caused by the direct connection of the ionic conductor (the ion-selective membrane) and the electron conductor (the electrode substrate) [1,3]. Twenty years later, this design was improved by using an intermediate layer between the ion-selective membrane and the substrate [3,4]. This group of electrodes is called solid-contact electrodes. The application of an intermediate layer improves the charge transport properties and eliminates the problem of potential instability [3]. So far, many materials have already been tested as intermediate layers, but new ones with better properties are still being sought.

Among the many requirements for materials used as intermediate layers, one of the most important is ion-to-electron conductivity [2,5,6]. Ion-to-electron conductors are characterized by redox or double-layer capacitance. The electrical capacity is a parameter of the material that allows one to easily compare the materials with each other. Also, in the case of modification of already-used materials, this parameter made it easy to determine whether the modification improved the electrical properties. Another important requirement for materials is their hydrophobicity [7]. The high hydrophobicity of the material prevents the formation of a thin layer of water at the interface between the solid contact and the membrane, which adversely affects the stability of the response. In addition, high hydrophobicity prevents delamination of the ion-selective membrane, extending the electrode’s lifetime.

Among groups of materials used as an intermediate layer, conductive polymers are one of the oldest [6,8]. Their structure makes them good charge transducers between materials with different types of conductivity [9]. Among the conductive polymers, one popularly used as an intermediate layer is poly(3-octylthiophene-2,5-diyl) (POT) [8,10,11]. It has good mediation properties with a relatively large value of the contact angle (approximately 90° [11]) compared to other conductive polymers. However, electrodes with a POT layer as a permanent contact are characterized by poor potential reproducibility and signal stability [10]. The properties of POT can be improved by using it as a composite material with nanosized ruthenium dioxide or molybdenum sulfide [11,12]. Among the materials previously modified by POTs are carbon nanotubes [13,14], which are elements of the hierarchical nanocomposites tested in this paper. Michalska’s group proposed the use of a nanocomposite material consisting of multi-walled carbon nanotubes and POT as a modifier of the surface of the GC electrodes [13], and Rostampur used an organic-base ink to construct a paper-based ISE by integrating single-walled carbon nanotubes and POT on filter paper used as a solid substrate for potentiometric sensors. In all cases, modification of intermediate materials allows one to improve their properties, and thus improve the performance of electrodes. In this work, we show whether the use of POT as a composite material is a universal solution and what impact it has on carbon materials with a more complex structure.

In our previous work, the use of electrospun carbon nanofibers and hierarchical nanocomposites as a permanent contact layer was presented [15]. Hierarchical nanocomposites are materials with a strictly designed architecture [16,17,18]. The tested materials were characterized by high hydrophobicity. The ready-to-use electrodes were characterized by high electrical capacity and stable response.

The aim of this work is to present the effect of a composite additive in the form of POT on the properties of the materials presented previously:

- Electrospun carbon nanofibers (eCNF);
- Electrospun carbon nanofibers with embedded cobalt nanoparticles (eCNF-Co);
- Hierarchical nanocomposite with the nanoparticles of cobalt and nickel as a catalyst for the growth of carbon nanotubes (eCNF/CNT-NiCo).

The combination of these materials with the POT allowed new materials with even higher hydrophobicity to be obtained and to improve electrical parameters in relation to the previously presented, pure materials.

## 2. Materials and Methods

### 2.1. Chemicals

The membrane components—potassium ionophore I (Valinomycin), lipophilic salt– potassium tetrakis(4-chlorophenyl)borate (KTpClPB), 2-nitrophenyl octyl ether (o-NPOE), and poly(vinyl chloride) (PVC)—were purchased from Sigma–Aldrich and dissolved in tetrahydrofuran (Sigma–Aldrich, Saint Louis, MO, USA).

Three different nanomaterials were tested as an intermediate layer in the construction of solid-contact electrodes. Dispersions of material in THF contained 5 mg/mL of the following materials: eCNF, eCNF-Co and eCNF/CNT-NiCo. The materials were prepared according to the method described by Zambrzycki [17]. These materials were studied in previous work, where their preparation was fully described [14]. Poly(3-octylthiophene-2,5-diyl) (POT) was purchased from Sigma–Aldrich.

Potassium chloride (KCl) was purchased from POCH (Gliwice, Poland), and solutions of K^+^ ions with concentrations from 10^−7^ to 10^−1^ M were used for potentiometry, chronopotentiometry, and EIS measurements.

### 2.2. Layers Preparations

The solid-contact-layer composite materials were prepared according to the method described by Lenar et al. [11]. Each of the composites was prepared on the basis of eCNF, eCNF-Co or eCNF/CNT-NiCo, respectively, modified with poly(3-octylthiophene-2,5-diyl) (POT) polymer. First, the carbon material (5 mg) and POT (10 mg) were ultrasonically dispersed in 1 mL of THF. Then the dispersion was centrifuged for 15 min (14,500 RPM). The solvent with the POT portion was separated from above the sediment. Then the solid part was redispersed in 1 ml of THF.

### 2.3. Preparation of SC-ISE

Solid-contact layers were casted onto glassy carbon disc electrodes (Mineral, Poland) using the drop casting method. The electrode area was 0.07 cm^2^. This method is fast, simple and inexpensive. At the beginning, glassy carbon disc electrodes were polished with alumina slurries of decreasing particle size (0.3 and 0.05 µm) and rinsed with water and methanol. Clean and dry electrodes were casted with 15 µL of solid-contact-layer solution. Layers were dried at room temperature. All electrodes were casted with 60 µL of membrane solution of a given composition: potassium ionophore I 1.10% (*w*/*w*), KTpClPB 0.25% (*w*/*w*), o-NPOE 65.65% (*w*/*w*), PVC 33.00% (*w*/*w*). Components of 0.25 g total weight were dissolved in 2 mL of THF. Three pieces of each electrode type were prepared, and all differed by composite material. Three coated disc electrodes served as a control group.

### 2.4. Measurements

The morphology of the materials tested as intermediate layers was characterized after synthesis using a scanning electron microscope (NOVA NANO SEM 200, FEI EUROPE COMPANY) with EDS analyzer (EDAX).

The wettability of each tested layer was determined by measuring the wetting angle using a Theta Lite contact angle microscope (Biolin Scientific, Gothenburg, Sweden) with One Attension software.

Electrochemical impedance spectroscopy and chronopotentiometric techniques were used to develop electrochemical properties of the studied materials. The measurements were performed using the Autolab General Purpose Electrochemical System (AUT302N.FRA-2-AUTOLAB, Eco Chemie, Utrecht, The Netherlands) that cooperated with the NOVA 2.1.4 software. Measurements were carried out in a three-electrode system with the single-junction Ag/AgCl reference electrode filled with 3 M KCl (6.0733.100 Ω Metrohm, Herisau, Switzerland), using a carbon glass rod as auxiliary and an indicator electrode with a particular layer.

Electrochemical impedance spectroscopy allowed us to determine the electrical capacity of the electrodes. Measurements were made in the frequency range of 100 kHz–0.01 Hz with an amplitude of 10 mV. The electrical capacity was determined from the dependence C = 1/(2πƒZ″) using the value of the imaginary part of an impedance at a frequency of 0.01 Hz. Chronopotentiograms were obtained according to the method proposed by Bobacka [3]. The potential of electrodes was registered during the forced current flow through the system. Current flow direction was changed after 60 s of measurements and potential were still registered. This allowed us to define three important parameters, such as electrical capacity (C = I(Δt/ΔEdc)), resistance (R = E/I) and potential drift (ΔE_dc_/Δt). All measurements of electrical characteristics were carried out in 10^−2^ M KCl solution.

Potentiometric measurements were made using a 16-channel EMF meter (Lawson Labs, Inc., Malvern, PA, USA) connected with a single-junction Ag/AgCl reference electrode filled with 3 M KCl (6.0733.100 Ω Metrohm, Herisau, Switzerland), a platinum auxiliary electrode and an all-solid-state electrode. The potentiometric response was recorded in KCl standard solutions of concentrations ranging from 10^−1^ to 10^−7^ M.

## 3. Results and Discussion

### 3.1. Microstructure Analysis by Scanning Electron Microscopy

**Figure 1:**
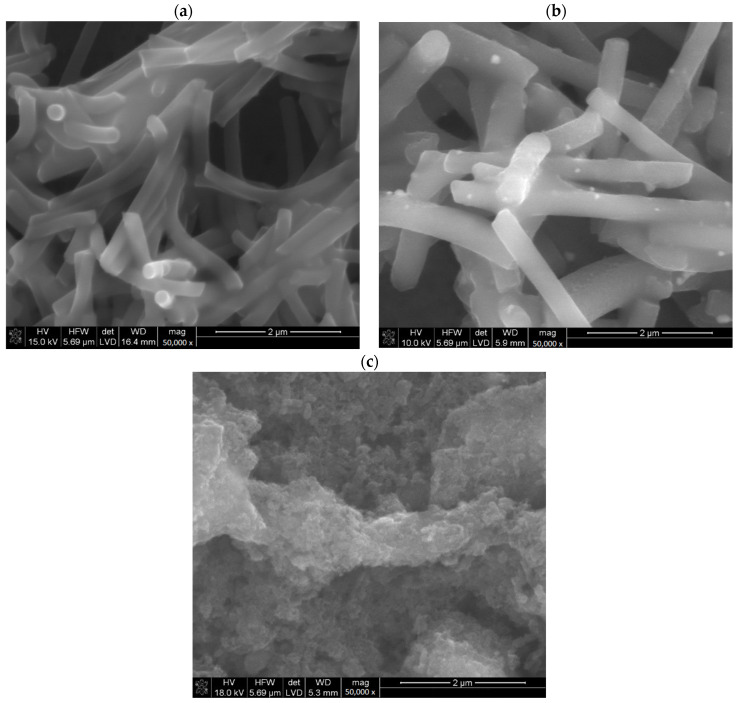
SEM miscroscans of investigated materials eCNF+POT (**a**), eCNF-Co+POT (**b**), eCNF/CNT-NiCo+POT (**c**).

Materials microstructures were characterized by scanning electron microscopy with energy-dispersive spectroscopy (EDS). In the microphotographs (Figure 1) it can be seen that POT is present in each prepared material and covers carbon materials. Micrographs of materials unmodified with POT were presented in our previous work [14].

The presence of POT in prepared materials for solid contact was confirmed by sulfur presence in EDS analysis (Table 1). The modification of each of the carbon materials was carried out successfully, and the POT evenly covers the modified material. The observed differences in the amount of sulfur in the tested layers result from the method of their preparation [11]. In the final product, POT is present only as a coating of the carbon material. The greater the surface development of the carbon material, the more polymer is present in the final composite material.

### 3.2. Wettablity

**Figure 2:**
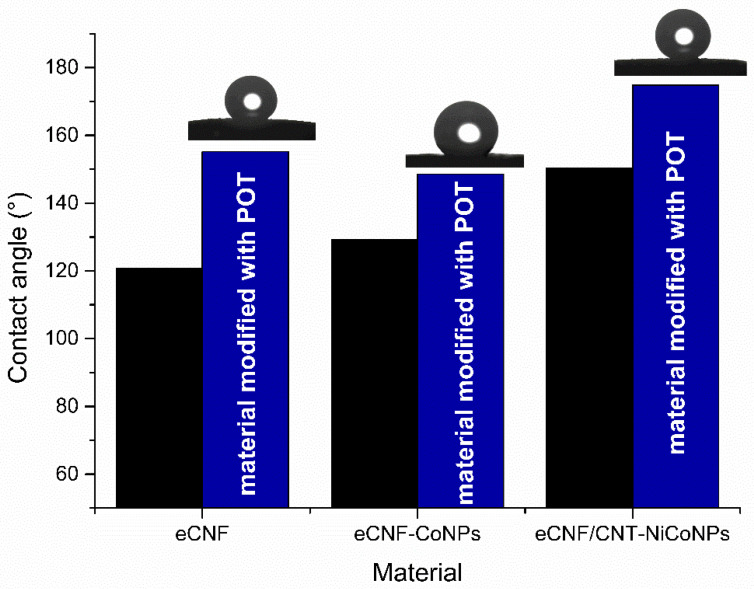
Contact angle values for POT-modified carbon materials (blue) compared to those previously published in [14] (black).

Hydrophobicity is one of the properties desirable in materials used as interlayers in ion-selective electrodes. High hydrophobicity prevents water from leaking under the ion-selective membrane and delaminating it from the electrode surface. The wettability test allows us to easily determine the hydrophobicity of a material by measuring the contact angle between the surface of the material and a drop of water dropped on it. Comparison of materials and photos obtained in the measurements are presented in Figure 2.

The modification of the material increased its hydrophobicity by 34° in the case of eCNF+POT (155°), 19° in the case of eCNF-Co3+POT (149°) and 24° in the case of eCNF/CNT-NiCo (175°). All obtained values are higher than those published for the sensors based on the mixture of carbon nanotubes and POT in paper-based sensors [13]. All tested materials were characterized by high hydrophobicity, which is favorable for their proposed use as an intermediate layer in solid-contact electrodes.

### 3.3. Electrical Characteristics of Electrodes

**Figure 3:**
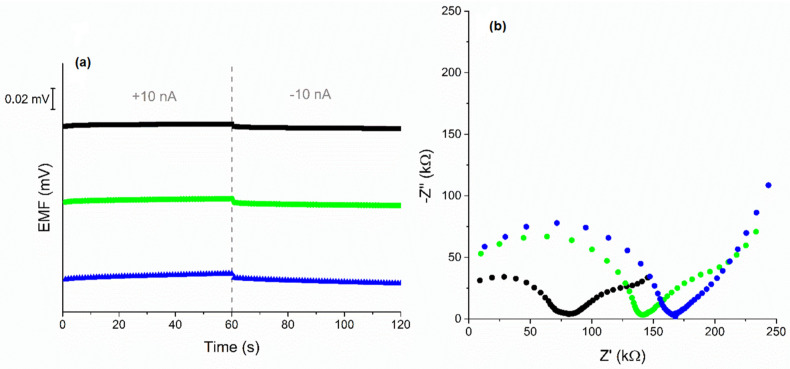
Chronopotentiograms of investigated electrodes (**a**) and Nyquist plots (**b**) of GC/eCNF+POT/K^+^-ISM (black), GC/eCNF-Co+POT/K^+^-ISM (green), GC/eCNF/CNT-NiCo+POT/K^+^-ISM (blue), recorded in 0.01 M KCl.

Ready-to-use electrodes with built-in tested materials were characterized in terms of electrical parameters by the method of chronopotentiometry. The electrode response was recorded during the forced current flow through the system. A current of 10 nA was used when the registration of potential changes at a lower current was not possible. Potential drift and electrical capacity were calculated based on line sections of recorded dependences E(t). In addition, the resistance of the electrodes was determined. The parameter values are summarized in Table 2, where they were compared with the results obtained for electrodes with unmodified materials. The obtained chronopotentiograms are presented in Figure 3a.

The electrical properties of the tested electrodes were also examined by electrochemical impedance spectroscopy. The results presented in the form of a Nyquist plot are shown in Figure 3b. In the high- and medium-frequency areas, two semicircles can be distinguished for the plots. The semicircles testify to the presence of materials with mediating properties—the ion-selective membrane and POT. The line in the low-frequency area corresponds to the characteristics of the processes taking place at the interface between the membrane and solid contact layer and allows for the determination of their electrical capacity. The obtained values are presented in Table 2. The relationship between the results obtained in the chronopotentiometry method and the EIS is well preserved. In addition, in the case of eCNF and eCNF-Co, modification with a polymer allowed significantly increased electrical charge capacity of the electrodes, which is a desired effect and explains the improvement of the potentiometric parameters of the tested electrodes.

In the case of the material eCNF/CNT-NiCo, the modification with POT resulted in a decrease in electrical capacity. Modification with polymer caused coverage not only of carbon nanotubes but also of the areas between them. This reduces the surface/volume ratio.

### 3.4. Potentometric Measurements

**Figure 4:**
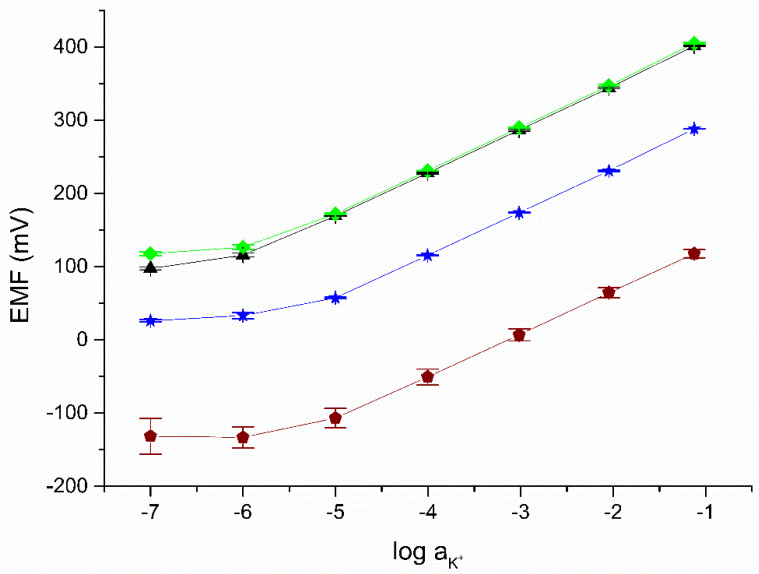
The potentiometric response recorded in KCl solutions for the following electrodes: GC/eCNF+POT/K^+^-ISM (black), GC/eCNF-Co+POT/K^+^-ISM (green), hierarchical nanocomposites GC/eCNF/CNT-NiCo+POT/K^+^-ISM (blue), and coated disc electrode (wine) (n = 3).

In the next stage, the ready-to-use solid-contact electrodes using the proposed materials as a mediation layer were calibrated. The dependence of the electromotive force on the log(a_K+_) determined for the tested electrodes was investigated based on calibration in solutions with a concentration of 10^−7^–10^−1^ M KCl on three consecutive days. The electrodes response recorded after 48, 72 and 96 h of conditioning in 0.01 M KCl is shown in Figure 4. The parameters of the calibration curves are summarized in Table 3. The slope determined on their basis is close to the Nernstian value for all tested electrodes.

The limit of detection was determined according to the method proposed by IUPAC as the activity of K^+^ ions at the intersection of the interpolated linear sections of the calibration curve. These are presented together with other metrological parameters in Table 3. The obtained values were compared with the parameters of the previously described solid-contact electrodes using mediation layer materials unmodified with POT. The electrode response in low-concentration solutions may be influenced by leakage of internal solution from the reference electrode.

From the results obtained, it can be seen easily that the electrodes show a stable response in the following days of measurement. The standard deviation of the normal potential for three calibrations on consecutive days for all tested electrodes is lower than for electrodes with unmodified material and calibrations performed consecutively. The stability of the response of the proposed electrodes may allow for an extension of the time between subsequent calibrations, which allows us to save time when performing chemical analyses.

### 3.5. Stability of Response and Reversibility Test

Another test performed to characterize the sensors consisted of examining the stability of the electrode’s response in a long-term study. The response of the electrodes was recorded in a 20-h measurement. Based on this, the potential drift as a derivative of the potential over time was determined. Leakage of internal solution from the reference electrode can affect potential drift, especially in a long-term stability test. In the test, a KCl solution with a concentration of 10^−2^ M was used, which minimizes the effect of electrolyte leakage. The results obtained showed that the modification of the material with a polymer made it possible to obtain electrodes with a stable response. The potential drift was 0.03 mV/h for eCNF+POT/K^+^-ISM, 0.03 mV/h for eCNF-Co+POT/K^+^-ISM and 0.06 mV/h for eCNF/CNT-NiCo+POT/K^+^-ISM. For comparison, the potential drift for the coated disc electrode in the same test was 0.8 mV/h. As previously reported, the potential drift for electrodes with unmodified materials was 0.09 mV/h for eCNF/K^+^-ISM, 0.17 mV/h for eCNF-Co/K^+^-ISM and 0.06 mV/h for eCNF/CNT-NiCo/K^+^-ISM [15]. 

In the case of carbon nanofibers and carbon nanofibers with Co nanoparticles, modification of the material with a polymer allows us to reduce the potential drift. The high stability of the electrode responses allows improvement in the accuracy of the results obtained with them and a reduction in the need for calibration.

**Figure 5:**
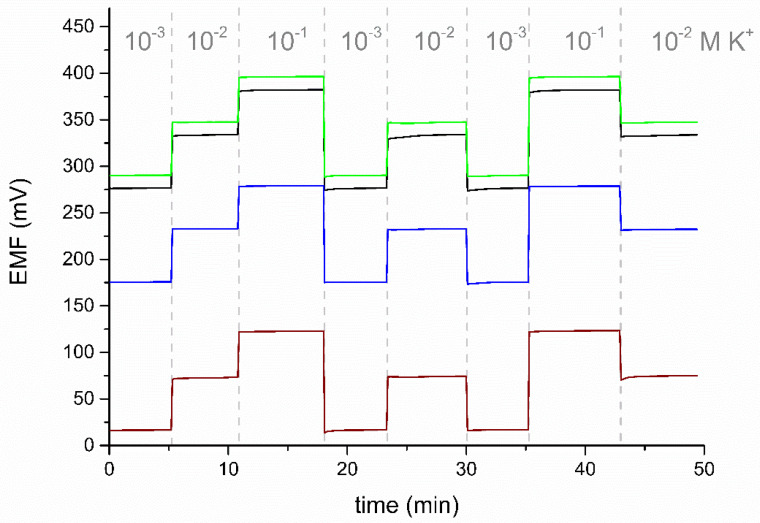
The reversibility test of electrodes GC/eCNF+POT/K^+^-ISM (black), GC/eCNF-Co+POT/K^+^-ISM (green), and GC/eCNF/CNT-NiCo+POT/K^+^-ISM (blue), and the coated disc electrode (wine), recorded in KCl solutions.

The tested electrodes show good potential reversibility (Figure 5). The value of the standard deviation for the potential recorded in a solution with a concentration of 10^−2^ M KCl is 0.2 mV for eCNF+POT/K^+^-ISM, 0.3 mV for eCNF-Co+POT/K^+^-ISM and 0.3 mV for eCNF/CNT-NiCo+POT/K^+^-ISM. The electrodes were characterized by a much better reversibility of the potential than the one obtained for the coated disc electrode, for which the standard deviation of the response in this solution was 0.7 mV.

## 4. Conclusions

In this work, the combination of two types of materials—carbon nanomaterials and a conductive polymer—was tested for application as a solid contact in solid-state electrodes. The research was conducted on an example of a potassium ion–selective membrane. Modification of the carbon materials with polymer (POT) is simple, fast and allows us to significantly improve the properties of the solid-contact layer. However, this is not a universal method. The addition of POT to electrospun carbon nanofibers increased the hydrophobicity by 34° and the capacitance of the solid-contact layer to 7.87 mF/cm^2^. The simultaneous increase of these two parameters is very desirable for applications in potentiometric sensors because it resulted in better metrological parameters of electrodes, including the stable and reversible response. In the case of the carbon nanofibers with the growth of carbon nanotubes material, despite the increase in hydrophobic properties, we did not observe an increase in the layer capacitance. All electrodes showed a response close to the Nernstian value. Moreover, a small standard deviation of the calibrations performed in the following days proved the reproducible response of the electrodes over time, making it possible to reduce the need for frequent calibrations.

## Figures and Tables

**Table 1:** Atomic content of sulfur, nickel and cobalt.

Material	S at%	Ni at%	Co at%
eCNF+POT	1.28	-	-
eCNF-Co+POT	2.27	-	1.68
eCNF/CNT-NiCo+POT	4.47	0.24	0.27

**Table 2:** Electrical parameters of the investigated K^+^-ISE.

Electrode GC/:	R ± SD [kΩ]	ΔE_dc_/Δt ± SD [μV/s]	C ± SD [mF/cm^2^] (CP Method)	C [μF] [mF/cm^2^] (EIS Method)
eCNF/K^+^-ISM [14]	91.3 ± 0.7	88.4 ± 1.3	1.62 ± 0.02	1.28
eCNF+POT/K^+^-ISM	113.8 ± 0.8	18.2 ± 0.6	7.87 ± 0.27	6.80
eCNF-Co/K^+^-ISM [14]	93.1 ± 0.1	53.5 ± 0.5	2.67 ± 0.03	1.79
eCNF-Co+POT/K^+^-ISM	179.7 ± 0.7	33 ± 0.4	4.37 ± 0.53	3.21
eCNF/CNT-NiCo/K^+^-ISM [14]	122.4 ± 0.7	31 ± 1.5	4.71 ± 0.14	3.80
eCNF/CNT-NiCo+POT/K^+^-ISM	197 ± 0	73 ± 3	1.97 ± 0.07	1.66

**Table 3:** Potentiometric parameters of the investigated K^+^-ISEs.

Electrode	Parameter			
	Slope (mV/dec)	E^0^ (mV)	LOD (M)	Linear Range (M)
GC/eCNF/K^+^-ISM [14]	59.45 ± 0.70	435.3 ± 1.7	10^−5.5 ± 0.1^	10^−5^–10^−1^
GC/eCNF+POT/K^+^-ISM	59.74 ± 0.2	467.9 ± 1.4	10^−6.2 ± 0.1^	10^−6^–10^−1^
GC/eCNF-Co/K^+^-ISM [14]	59.32 ± 0.80	401.0 ± 1.7	10^−5.7 ± 0.1^	10^−5^–10^−1^
GC/eCNF-Co+POT/K^+^-ISM	59.87 ± 0.2	470.9 ± 1.3	10^−5.9 ± 0.1^	10^−5^–10^−1^
GC/eCNF/CNT-NiCo/K^+^-ISM [14]	59.39 ± 0.80	413.7 ± 0.9	10^−6.3 ± 0.1^	10^−6^–10^−1^
GC/eCNF/CNT-NiCo+POT/K^+^-ISM	59.76 ± 0.3	354.8 ± 0.8	10^−5.5 ± 0.1^	10^−5^–10^−1^
GC/K^+^-ISM	57.0 ± 2.0	178.6 ± 3.1	10^−5.3 ± 0.2^	10^−5^–10^−1^

## Data Availability

Not applicable.
